# The Determination of Radon/Thoron Exhalation Rate in an Underground Coal Mine—Preliminary Results

**DOI:** 10.3390/ijerph19106038

**Published:** 2022-05-16

**Authors:** Michał Bonczyk, Stanisław Chałupnik, Malgorzata Wysocka, Agata Grygier, Robert Hildebrandt, Zornitza Tosheva

**Affiliations:** 1 Główny Instytut Górnictwa, Plac Gwarków 1, 40-166 Katowice, Poland; schalupnik@gig.eu (S.C.); mwysocka@gig.eu (M.W.); agrygier@gig.eu (A.G.); rhildebrandt@gig.eu (R.H.); 2 Department of Physics, University of Luxemburg, 2 Av. de l’Universite, 4365 Esch-sur-Alzette, L-5610 Luxembourg, Luxembourg; zornitza.tosheva@gmail.com

**Keywords:** radon, thoron, exhalation, underground coal mine

## Abstract

The objective of this work was to perform a series of measurements of radon and thoron exhalation in the underground workings of an experimental coal mine. In the years 2012–2015, experiments on underground coal gasification were carried out in a coal mine, which caused, among other effects, damage to rock mass. Afterward, periodic increases in the concentration of potential alpha energy (PAEC) of radon decay products in the air were found, which could pose a hazard to miners. The question posed was whether the gasification experiment resulted in the increased migration of radon and thoron. If so, did it increase the radiation hazard to miners? The adaptation of the existing instrumentation to the specific conditions was conducted, and a series of measurements were made. It was found that the measured values of radon and thoron exhalation rates ranged from 3.0 up to 38 Bq·m^−2^·h^−1^ for radon and from 500 up to 2000 Bq·m^−2^·h^−1^ for thoron.

## 1. Introduction

Several years ago, hard coal underground gasification for hydrogen production experiments were performed in two collieries in the Upper Silesia Coal Basin (USCB), Poland. One of these collieries was a shallow, experimental mine, with a depth not exceeding 50 m, while another one was an active mine. Preliminary measurements of radon revealed that in the galleries of the experimental mine, very high radon levels were found, reaching 30–43 kBq·m^−3^, while the maximum radon concentration measured in the other coal mine reached 15 kBq·m^−3^ [1] Additionally, sudden changes in concentration were observed, although no correlation with atmospheric pressure changes was found. The authors decided to adopt the accumulation chamber, designed for measurements of radon exhalation rate from the soil, to perform measurements of radon and thoron exhalation rates from the walls of underground galleries. It became necessary to apply active monitors, allowing not only radon but also thoron gas concentrations to be monitored. For this purpose, RAD7 and AlphaGuard were used to measure not only exhalation rates but also radon and thoron gas concentrations in the ventilation air. Additionally, the PAECs of radon and thoron decay products were measured with the use of ALFA probes, equipped with thermoluminescent detectors (TLDs).

The hazard caused by radon isotopes (^222^Rn, ^220^Rn) is due to the emission of gaseous radionuclides from solid materials (soil, rocks, and construction materials) or water into confined spaces in dwellings or underground galleries [2,3,4,5,6,7,8,9,10,11,12,13,14]. The first stage of radon migration from a crystalline lattice is emanation. This phenomenon can be described as radon gas entry into the air in the pore space of solid materials due to the recoil effect. The emanation coefficient is applied for this purpose, describing the ability of radon atoms to leave grains of solid material in relation to the total number of radon atoms. Radon is produced as the effect of radium decay (^226^Ra or ^228^Ra) in a solid material [15]. The recoil energy is crucial for the value of the emanation factor. In the case of ^222^Rn, the recoil energy of a produced atom (0.086 MeV) may cause movement in minerals within the range of 20–70 nm only; for thoron, these values are similar. As a result of the emanation effect, radon and thoron are present in the pore air in the dynamic equilibrium with radium isotopes in the solid phase. Afterward, radon can be transported in pore spaces and further along the fissures and cracks in rock bodies or soil. The main processes responsible for transport are diffusion and advection. In the next step of migration, radon isotopes may leave the material. Parts of radon atoms from pore space can diffuse out from the solid material into the atmosphere, and this phenomenon is called exhalation. According to Nazaroff [15], the exhalation coefficient can be calculated from the following formula:

(1) $$\varphi \mathrm{Rn}=\varepsilon \cdot A_{\mathrm{rad}}\cdot \lambda \cdot R_{B}\left[\mathrm{Bq}\cdot m^{-2}\cdot s^{-1}\right]$$

where ε—emanation coefficient; A_rad_—radium content in the soil; ρ—density of the soil; λ—decay constant of radon; R_B_—diffusion length of radon. Another option is to measure radon exhalation from the ground or construction materials. The exhalation coefficient from the soil was assessed under normal conditions by Wilkening as 17 mBq·m^−2^·s^−1^ [16]. Porstendorfer [17] calculated the average exhalation rate from the soil as 26 mBq·m^−2^·s^−1^. On the other hand, Colle [18] measured the exhalation coefficient within the range of 2 to 50 mBq·m^−2^·s^−1^. Our measurements in the Upper Silesia region showed results between 1 and 500 mBq·m^−2^·s^−1^ [19,20].

Due to exhalation from the gobs and walls of underground galleries, in confined spaces, the radon level can be significantly enhanced in comparison with atmospheric air. During our investigations in the USCB, it was revealed that radon levels in the mines were sometimes much higher than the radon concentration outdoors or even in dwellings, especially in the vicinity of exploited zones [21]. Additionally, in some parts of the USCB, radon concentrations in dwellings were enhanced, too [19]. The main reason for this situation is that underground exploitation induces the creation of emptiness, cracks, and fissures in the strata. All of the factors mentioned above very often cause subsidence of the surface and damage to buildings located in affected zones. Radon migration in fractured rocks and soil happens much more often than in undisturbed strata [19].

Proper measurements of thoron and the potential alpha energy concentration (PAEC) in decay products are not easy to perform, especially in the case when both radon isotopes (^222^Rn and ^220^Rn) are present in the environment. Due to its very short half-life (55.6 s), ^220^Rn decays quickly, and variations of its concentration in the air (atmosphere, indoors, and soil gas) are significant. Therefore, it is difficult to estimate the dose due to ^220^Rn and decay products’ inhalation, making the estimation of the dose unreliable. Many efforts have been undertaken by numerous groups of scientists to solve these problems [14,22,23,24,25,26,27,28,29,30]. The estimation of the average ^220^Rn concentration in an indoor environment is often based on measurements of thoron exhalation rates, further converted into the average concentration of thoron in the air. However, the dose estimation requires calculations of decay products’ PAEC with a proper equilibrium factor F. Finally, the inhalation dose should be assessed with the application of appropriate dose conversion factors.

In Poland, it has been found that the majority of the committed effective dose in collieries was related to radon’s short-lived progeny, not radon itself. Therefore, Polish regulations are focused on measurements of the potential alpha energy concentration of the radon progeny (PAEC) instead of radon gas [31]. It should be pointed out that the determination of all short-lived radon progeny activity concentrations in mine air is rarely used to evaluate risk assessments in underground non-uranium mines [32]. It has been found that the equilibrium factor between radon and its short-lived progeny in underground coal mine atmospheres was very unstable and varied in space and time in the range of 0.05–0.95 [21]. Therefore, it was decided to measure the potential alpha energy concentration (PAEC) of the radon progeny directly to avoid uncertainties due to the approximation of the exposure from radon data [21,32]. Similar problems were also reported by Page and Smith in UK coal mines [33]. The highest value of the PAEC ever measured in Polish coal mines was equal to 63 μJ·m^−3^. Such a PAEC value was observed in the Lower Silesian Basin, in one of the coal mine galleries located close to old uranium works. During the last 25 years, in active collieries of the Upper Silesian Coal Basin, the PAEC values never exceeded 15 μJ·m^−3^ [34,35,36]. Many decades of investigations conducted in uranium and non-uranium mines show that the presence of privileged pathways of radon migration in the strata was correlated with enhanced levels of radon in the ventilation air [21]. An overview of investigations performed in uranium mines [37,38] showed that the porosity of ore-bearing rocks was an important factor, although smaller variability was found for porous rocks.

## 2. Materials and Methods

### 2.1. Study Area

The study was carried out at the Experimental Mine (EM) ‘Barbara’, Poland, which is located in the central part of the Upper Silesian Coal Basin (USCB). This mine is a part of the Central Mining Institute. At EM ‘Barbara’, the underground workings were adapted to the requirements of our investigations. At present, the underground infrastructure enables the performance of exhalation experiments.

**Figure 1:**
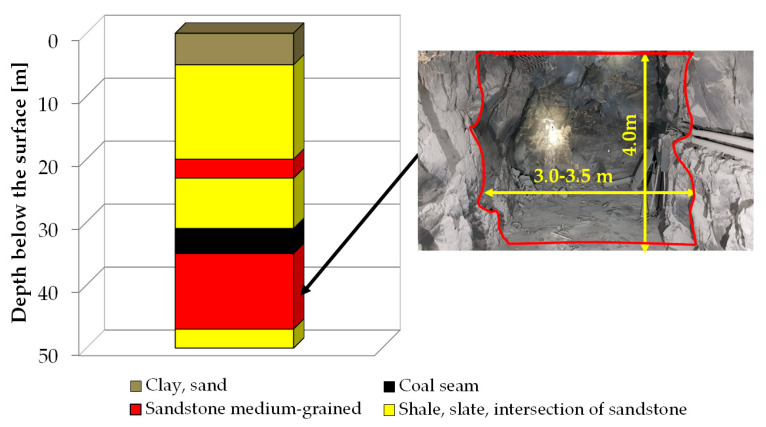
Stratigraphic profile of the GIG Experimental Mine ‘Barbara’.

The EM ‘Barbara’ area is located in the main anticline of the USCB. The geological structure of the deposit consists of Quaternary and Productive Carboniferous strata. Quaternary formations are sand, gravel, and clay. In a major part of the area of interest, the thickness of the Quaternary varies from 4 m to 6 m. Productive Carboniferous strata is represented by the Orzeskie and Łaziska Beds. The Łaziska Beds are formed from sandstone–conglomerate facies, interlaid with shale. The Orzeskie Beds are formed from fine- and medium-grained sandstones, conglomerate, shales, and numerous hard coal beds. The rock mass within the zone of the mining works is not tectonically disturbed. The simplified vertical cross-section of the strata is shown in Figure 1. It can be seen that under Quaternary cover, there are Carboniferous rocks and coal seams.

Several years ago, underground experiments of underground coal gasification (UCG) for hydrogen production were performed in two collieries in Poland [39]. One of these collieries (EC ‘Barbara’) was a shallow, experimental mine, with a depth not exceeding 50 m. The UCG reactor was drilled in the coal bed (−30 m), while galleries chosen for our investigation were located below at a level of −46 m—see Figure 1, cross section and photography. These experiments caused the creation of cavities after coal was burnt out, but additionally, the high temperature and pressure affected the strata in the vicinity. These pathways would allow radon ^222^Rn migration for relatively long distances. On the other hand, due to the short half-life, thoron ^220^Rn would migrate a very short distance, in spite of the presence of such pathways. After the exhalation of radon and thoron into air in the galleries, their decay led to the appearance of their short-living decay products—^218^Po, ^214^Pb, ^214^Bi, and ^214^Po for radon and ^216^Po, ^212^Pb, ^212^Bi, ^212^Po, and ^208^Tl for thoron. These radionuclides are the real sources of radiation hazards; therefore, the concentration of so called potential alpha energy (PAEC) is monitored to assess the radiation risk for miners.

The historical data of potential alpha energy concentration (PAEC) measured by mine staff are shown in Table 1.

The observed values of PAEC indicated the elevated levels of radon concentration. This motivated the initiation of the measurements of parent nuclides of decay products, meaning radon and thoron themselves. The assumption was made that the observed high variability in PAEC values was caused by significant variability in the exhalation rates of radon from walls, roofs, and bottoms. Additionally, we decided to measure not only radon but thoron exhalation as well to find specific patterns of their behavior.

### 2.2. Measurement of Radon and Thoron

**Figure 2:**
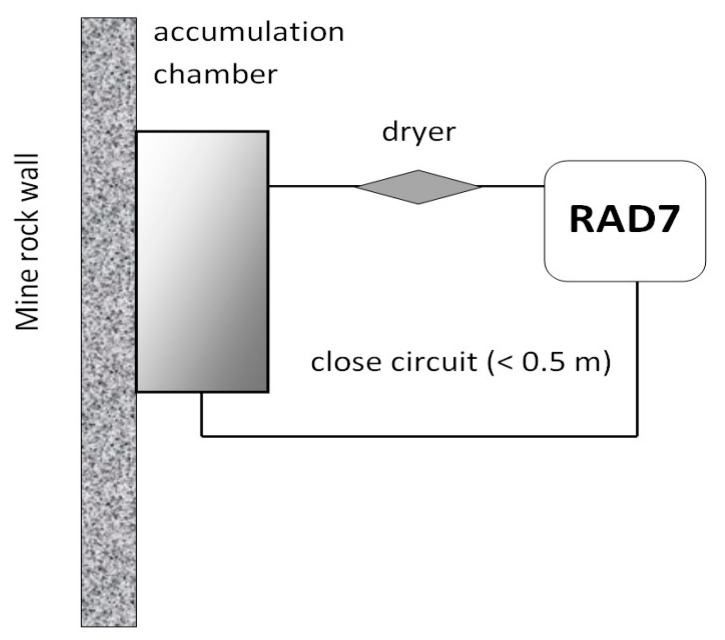
Method of measurement of radon and thoron exhalation from the soil with an application of the accumulation chamber.

**Figure 3:**
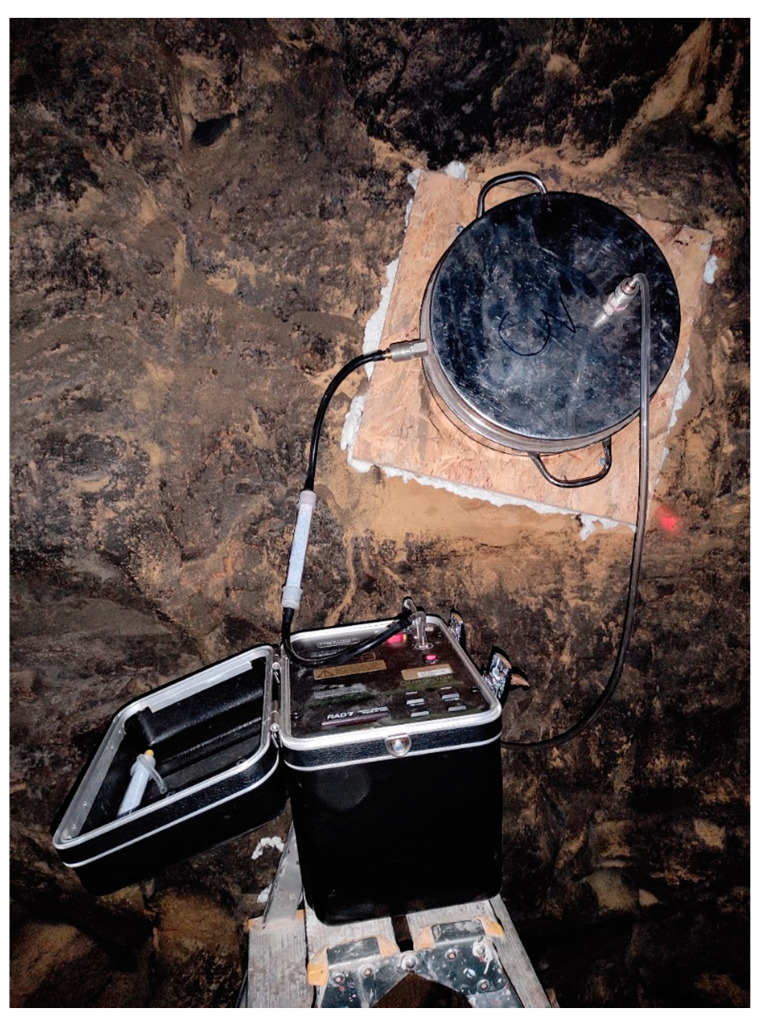
Measurement of radon and thoron exhalation rate in an underground gallery.

The monitoring of radon exhalation from soils in areas affected by mining began in Poland a few decades ago [4,19]. A similar method was applied for measurements of radon and thoron exhalation rates in underground mines. An accumulation was constructed for these measurements. The sketch picture of the chamber is shown in Figure 2. We applied a metal cylinder with a diameter of 350 mm and a height of 150 mm. The bottom edge of the cylinder was fixed to the wall and sealed with special adapter (Figure 3) in order to prevent ventilation via ambient air during measurements [40]. Two connectors (Swagelok, Solon, OH, USA) were used to enable air sampling from the chamber. To avoid overpressurizing the chamber and the back diffusion of radon, one of the valves was always opened during the accumulation time.

The application of the RAD7 radon/thoron monitor for thoron exhalation rate measurements was described by Tan and Xiao [41]. Investigations were performed with the use of the accumulation chamber technique and RAD7 as a radon/thoron detector. In the opinion of the authors, thoron’s rapid decay caused the inlet design and the airflow rate to become important factors in the calibration of the system. The method was applied for measurements of the thoron and radon exhalation rates. The authors decided to adopt the accumulation chamber, designed for measurements of radon exhalation rate from the soil, to perform measurements of radon and thoron exhalation rates from the walls of underground galleries. It became necessary to apply active monitors, allowing not only radon but also thoron gas concentrations and temporal changes in the emissions to be monitored. For this purpose, RAD7 was used to measure not only exhalation rates but also radon and thoron gas measurements in the ventilation air. The ‘Sniff’ mode was applied for the simultaneous measurement of radon and thoron concentration. The calibration of the RAD7 monitor was performed in the radon/thoron chamber. The reference values of radon/thoron concentration during calibration were determined using the liquid scintillation method. This method was described by Chałupnik [42].

Generally, changes in radon concentration under an accumulation chamber are described by exponential function:

(2) $$C\left(t\right)=C_{0}+C_{m}\left(1-e^{-\lambda t}\right)$$

where:

*λ*—decay constant of radon/thoron (h^−1^);

*C*_0_, *C_m_*—radon concentration in the accumulation chamber at time = 0 and the maximum value (Bq·m^−3^).

The initial part of the above exponential function can be approximated by the linear function. In order to avoid the long measurement of saturation concentration *C_m_*, the linear growth, visible at the beginning of the measurement, was analyzed. An additional advantage of such an approach was the effect of back diffusion was avoided in the case of a higher radon concentration under the accumulation chamber.

The exhalation rate can be calculated from:

(3) $$E_{Rn}=\frac{dC}{dt}\cdot \frac{V}{S}$$

where:

*E_Rn_*—(Bq·m^−2^·h^−1^);

*dC*/*dt*—growth in radon concentration under chamber (Bq·m^−3^·h^−1^);

*V*—the volume of the chamber (m^3^);

*S*—exhalation surface (m^2^).

In the case of thoron, due to the short half-life (55.6 s), the saturation was reached within several minutes, and growth was not visible if the sampling time was 20–30 min. In order to calculate the exhalation rate, the following formula was used:

(4) $$E_{Tn}=\frac{V\cdot \lambda_{Tn}}{S}\cdot C_{m}$$

where:

*E_Tn_*—exhalation rate (Bq·m^−2^·h^−1^);

*C_m_*—saturation concentration of thoron (Bq·m^−3^);

*λ*—decay constant of thoron (h^−1^);

*V*—the volume of the chamber (m^3^);

*S*—exhalation surface (m^2^).

### 2.3. Measurements of Radium and Thorium Concentration in Rocks

The activity concentrations of the following radionuclides: ^226^Ra, ^228^Ra, ^228^Th, and ^40^K, were determined using a gamma-ray spectrometer, equipped with a hyper pure germanium detector (HPGe) with the relative efficiency of 35%, cooled by liquid nitrogen (LN_2_). The energy calibration was performed using a multi-gamma source that contained isotopes emitting gamma lines in a wide range (46–2000 keV). The efficiency calibration was performed with the use of standard sources made of reference materials RGU-1, RGTh-1, and RGK-1, delivered by the International Atomic Energy Agency (IAEA). The following gamma spectra lines were used:

^226^Ra determined directly (186.2 keV—including interference with 185.7 keV from ^235^U) as well as via short-lived radon progeny ^214^Bi (609, 1120, 1765 keV) and ^214^Pb (295, 352 keV);

^228^Ra determined via ^228^Ac (911 keV);

^228^Th determined via ^212^Pb (239 keV) and ^208^Tl (583 keV), including correction related to the branching of decay chain;

^40^K determined directly (1461 keV).

Three samples of rock (sandstone) were collected from site where radon and thoron measurements were conducted.

## 3. Results

Measurements of radon and radon decay products’ PAEC revealed that in galleries of the experimental mine, very high radon levels were found. Due to the fact that the mine is an experimental one, coal is not exploited there; however, Polish regulations require the periodic measurements of PAEC of radon decay products during one shift at least once per three months. There are no legal requirements [32] to measure radon concentrations in mines, as the equilibrium factor between radon and decay products can vary in a very wide range. Therefore, a radon-based risk assessment is not necessary. The results of the long-term monitoring are presented in Table 1.

**Figure 4:**
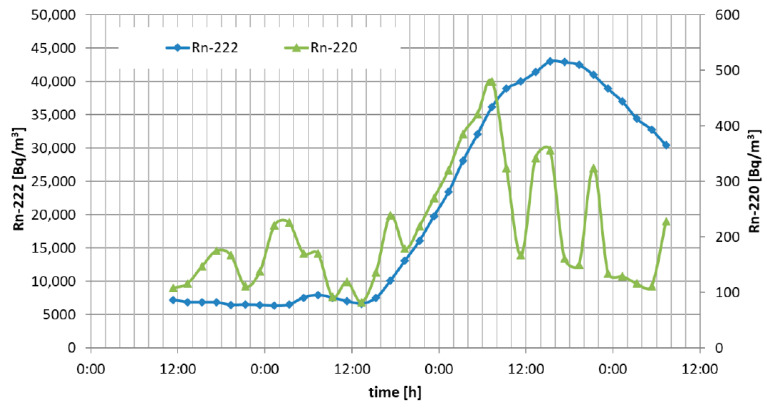
Results of radon (orange) and thoron (green) concentration in the gallery—1st campaign.

**Figure 5:**
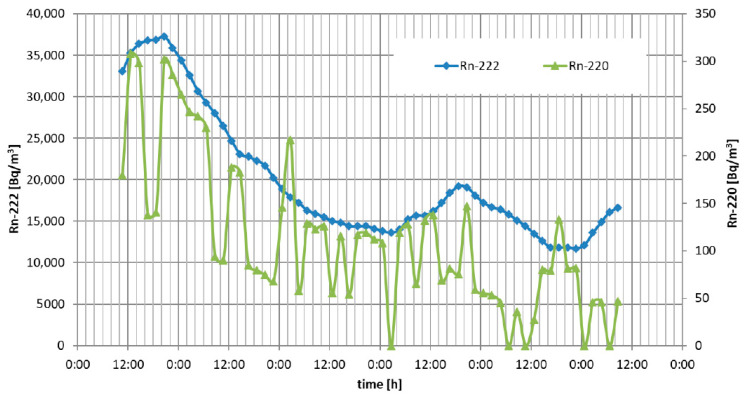
Results of radon (orange) and thoron (green) concentration in the gallery—2nd campaign.

The continuous measurement of radon and thoron air concentration in the experimental colliery at the gallery level of −46 m was performed. The results of two measurement campaigns are shown in the charts in Figure 4 and Figure 5.

During the experiments, the ventilation system was turned off and the gallery was sealed with the use of ventilation dams. We did not observe any correlation between changes in radon/thoron concentration and measured atmospheric air parameters such as pressure or temperature. In general, the seven measurement campaigns were performed within 5 months in 2021. Campaigns took from 72 to 120 h. The measured concentration varied from several hundred up to 43,000 Bq·m^−3^ for radon and from 80 up to 500 Bq·m^−3^ for thoron.

**Figure 6:**
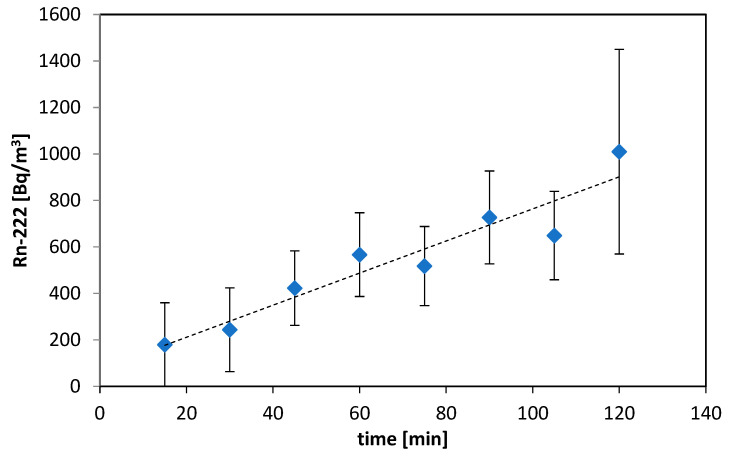
Ingrowth of radon concentration under accumulation chamber.

**Figure 7:**
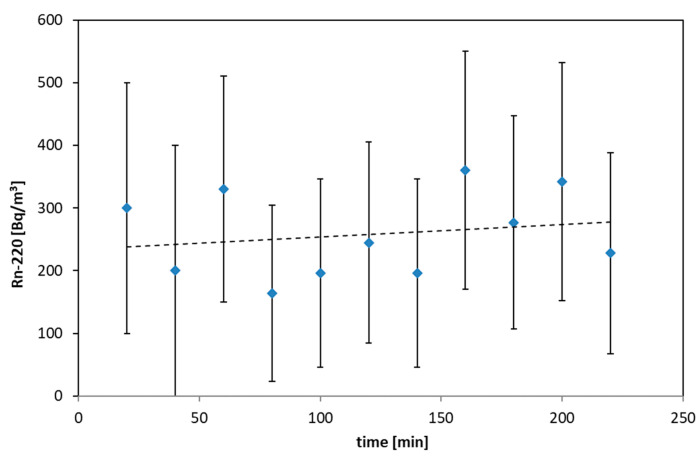
Thoron concentration under accumulation chamber.

The set of results of radon and thoron exhalation measurements are shown in Figure 6 and Figure 7. The total expanded uncertainty is shown in the charts in Figure 6 and Figure 7.

The duration of each test with the use of an accumulation chamber was 3–4 h. In general, 20 tests of exhalation rates were performed. The accumulation chamber was placed on the bottom, walls, and the roof of the gallery.

In the case of thoron, the growth was not visible due to the experimental conditions. Due to the half-life of thoron (55.6 s) and volume of the chamber, the saturation concentration of thoron was measured. Hence, no growth but a relatively stable level of thoron concentration was visible.

Based on fitted line and regression parameters for radon and average concentrations for thoron, the exhalation rates were calculated. The obtained exhalation rates varied from 3.0 up to 38 Bq·m^−2^·h^−1^ for radon and from 500 up to 2000 Bq·m^−2^·h^−1^ for thoron.

Sudden changes in concentration were observed, although no correlation with atmospheric pressure was found. The concentrations of radium (^226^Ra) and thorium (^228^Th) measured in samples collected from surrounding rock body were at average levels for the Earth’s crust—about 12 Bq·kg^−1^ of ^226^Ra and 13 Bq·kg^−1^ of ^228^Th. The exact results of gamma-ray spectrometry measurements are shown in Table 2. This means that apparently, the only reason for the high radon exhalation was extensive damage to the rock mass due to UCG reactor presence and high temperature and overpressure during the process of coal gasification.

## 4. Conclusions

The instrumentation for the measurements of radon and thoron exhalation rates and concentrations in ventilation air was chosen and adopted to allow monitoring to take place inside the shallow colliery in the vicinity of the former UCG reactor.

Preliminary measurements showed that a significant increase in radon levels was observed, as we had suspected. It is possible that this phenomenon is related to the vicinity of the former UCG reactor, where experiments on underground coal gasification were performed several years earlier. Probably, due to high temperature and pressure during that process, the strata underwent a transformation—a lot of cracks and fissures appeared, the porosity rose, and the radon migration became much more common. However, many more experiments and the accurate analysis of the data will be necessary to find an explanation for the phenomenon of the periodical increase in radon concentration up to 43 kBq·m^−3^. In particular, differences in radon and thoron exhalation rates will be useful in finding the reason for such behavior.

An unexpected problem is a high potential radiation risk for people involved in experiments or regular work in the vicinity of the underground reactor for coal gasification UCG. This issue must be taken into consideration in the future, despite the fact that this problem was never pointed out earlier.

According to Polish regulations, there is no radiation hazard in the mine (gallery) if the PAEC concentration is below 0.5 μJ·m^−3^. It can be seen that every year (except 2019), the monitoring results were above that value at a horizon of −30 m. It means that the crew members working there 1800 h per year could receive a dose exceeding 1 mSv·y^−1^. Moreover, when the PAEC exceeds 2.5 μJ·m^−3^, the annual dose might be higher than 6 mSv, and such a place should be treated as a controlled class A area; therefore, personal dosimetry must be applied for the permanent staff there. There is no permanent staff in this gallery, although a potential risk for miners exists.

Preliminary data from previous investigations confirmed that until now, no correlation between parent radionuclides concentrations in the strata (^226^Ra and ^228^Ra) and exhalation rates from galleries’ walls was found. Moreover, no connections between exhalation rates and atmospheric pressure or temperature in the galleries were observed.

It has been preliminarily stated that significant changes were observed in a correlation with water pumping from the strata, conducted for the dewatering of the mine. This means that the main source of radon was most likely the strata damaged during coal gasification experiments. However, it will be necessary to conduct more experiments in this place and nearby to find any relationship between these facts.

## Figures and Tables

**Table 1:** Results of PAEC monitoring in the experimental mine—values in μJ·m^−3^.

Date	Ventilation Shaft, Level −30 m		Ventilation Duct, Level −46 m	
	Average PAEC	PAEC Range	Average PAEC	PAEC Range
2012	1.60 ± 0.49	0.39–3.66	0.39 ± 0.23	<0.10–0.79
2013	0.98 ± 0.49	<0.1–1.56	<0.1	<0.1
2014	0.47 ± 0.15	0.32–0.68	0.21 ± 0.10	<0.1–0.34
2015	1.06 ± 0.24	<0.1–1.92	0.31 ± 0.16	<0.1–0.89
2016	0.29 ± 0.15	<0.1–0.69	0.15 ± 0.10	<0.1–0.26
2017	0.69 ± 0.19	0.37–1.01	0.13 ± 0.10	<0.1–0.24
2018	0.46 ± 0.14	0.15–0.76	0.27 ± 0.12	<0.1–0.38
2019	0.40 ± 0.15	0.34–0.46	0.11 ± 0.10	<0.10–0.14
2020	0.36 ± 0.14	0.31–0.80	0.13 ± 0.10	<0.10–0.16
2021	2.31 ± 1.45	0.35–7.36	0.55 ± 0.30	<0.10–1.25

**Table 2:** The concentration of radionuclides in rocks.

Sample No.	^226^Ra	^228^Ra	^228^Th	^40^K
	Bq·kg^−1^			
1	14.1 ± 1.3	15.9 ± 1.5	16.0 ± 1.3	413 ± 34
2	9.0 ± 0.7	10.4 ± 1.1	10.4 ± 1.1	483 ± 38
3	12.4 ± 1.1	14.8 ± 1.5	14.0 ± 0.9	438 ± 34

## Data Availability

Analyses in this study were based on existing data of radon concentration in dwellings. The measurements were performed by the authors of the paper and gathered in our database. Data sharing is not applicable to this article.
